# Thermal Quenching Mechanism of Mn^4+^ in Na_2_SiF_6_, NaKSiF_6_, and K_2_SiF_6_ Phosphors: Insights from the First-Principles Analysis

**DOI:** 10.1021/acs.inorgchem.4c03589

**Published:** 2024-10-21

**Authors:** Mekhrdod S. Kurboniyon, Alok M. Srivastava, Bibo Lou, Dilshod D. Nematov, Amondulloi Burhonzoda, Tomoyuki Yamamoto, Chong-Geng Ma, Mikhail G. Brik

**Affiliations:** 1 School of Optoelectronic Engineering & CQUPT-BUL Innovation Institute, Chongqing University of Posts and Telecommunications, Chongqing 400065, China; 2 National Academy of Sciences of Tajikistan, Dushanbe 734025, Tajikistan; 3 Kagami Memorial Research Institute for Materials Science and Technology, Waseda University, Tokyo 169-0051, Japan; 4 Current Lighting Solutions LLC, 1099 Ivanhoe Road, Cleveland, Ohio 44110, United States; 5 Physical−Technical Institute, National Academy of Sciences of Tajikistan, Dushanbe 734063, Tajikistan; 6 Centre of Excellence for Photoconversion, Vinča Institute of Nuclear Sciences - National Institute of the Republic of Serbia, University of Belgrade, Belgrade Serbia; 7 Institute of Physics, University of Tartu, W. Ostwald Str. 1, Tartu 50411, Estonia; 8 Academy of Romanian Scientists, 3 Ilfov, Bucharest 050044, Romania

## Abstract

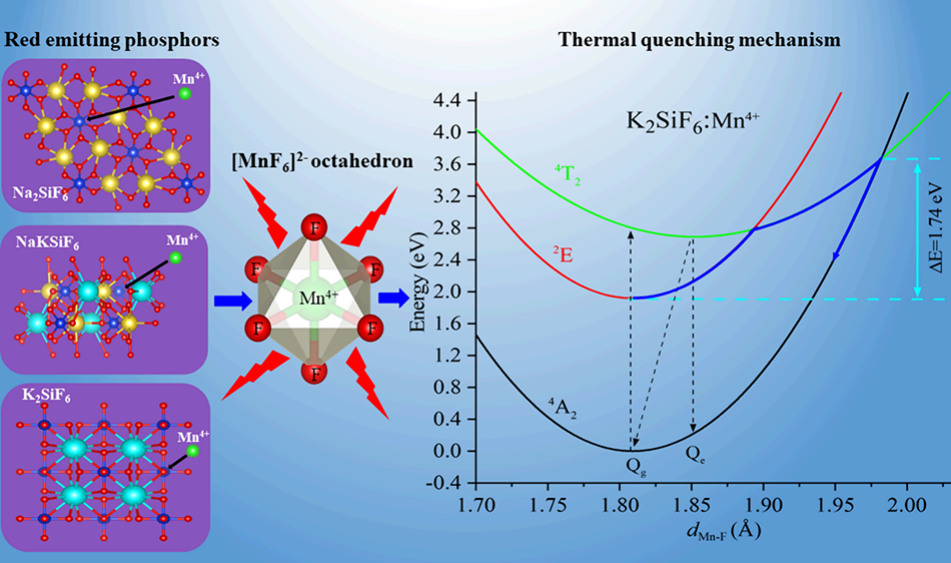

This study aims to identify the key factors governing the thermal quenching of Mn^4+^ ion luminescence in fluoride-based phosphor materials used as red emitters in modern-day phosphor-converted LED devices. Here, we employ first-principles calculations for Mn^4+^-doped Na_2_SiF_6_, NaKSiF_6_, and K_2_SiF_6_ hosts to explore how host properties and local coordination environments influence thermal quenching behavior. The ΔSCF method was used to model the geometric structures of the Mn^4+4^A_2_ (ground) and ^2^E, ^4^T_2_ (excited) states and the energies of the optical transitions between these states. Our results reveal that thermal quenching in Na_2_SiF_6_ and K_2_SiF_6_ phosphors occurs through thermally activated ^2^E → ^4^T_2_ → ^4^A_2_ crossover. In contrast, thermal quenching in NaKSiF_6_ is due to other nonradiative decay pathways. Investigations of the mechanical stability of these fluorides show that NaKSiF_6_ is mechanically unstable. We suggest that this property of the host limits the luminescence efficiency of the embedded Mn^4+^ ions. We also determined the reason for the difference in the intensity of the ^2^E → ^4^A_2_ emission transition (ZPL) in the systems. These findings advance our fundamental understanding of the thermal quenching mechanism of Mn^4+^ ion luminescence in fluorides, and the results can aid future discoveries of technologically useful phosphors through high-throughput design methodologies.

## Abstract 2

Schematic representations for the crystal structure of Na_2_SiF_6_, NaKSiF_6_, and K_2_SiF_6_ compounds together with the local coordination environments of the [MnF6]^2−^ octahedron in K_2_SiF_6_. Configuration coordinate diagrams showing luminescence quenching due to a thermally activated crossover via the Mn^4+^^4^T_2_ excited state. The ^4^A_2_ ground state energy is used as the reference point (zero). Δ*E* represents the calculated activation energy for thermal quenching.

## 1 Introduction

The electronic transitions between the energy levels of the unfilled 3d shell of transition metal ions (TM) manifest themselves in the form of intense and broad (or sharp) luminescence bands. The nature of these bands depends on the spin and symmetry of the involved states and on the dynamic coupling with the local environment surrounding these ions within the host lattice. The luminescence of TM with the 3d^3^ electronic configuration, such as Mn^4+^ and Cr^3+^, is currently under intense investigations as commercial, residential, and specialty lighting rapidly shifts toward phosphor-converted light-emitting diodes (pc-LEDs). Red emitting phosphors for LED lighting have been the subject of intense research, requiring both strong absorption at 450 nm and narrow line emission around 630 nm with minimal emission beyond 650 nm for optimal efficacy. The K_2_SiF_6_:Mn^4+^ (KSF) phosphor satisfies the various requirements of a red emitter for white light pc-LED. The phosphor has been commercially implemented in pc-LEDs for general illumination and display devices. Recently, the Na_2_SiF_6_:Mn^4+^ (NSF) phosphor has been commercialized. The commercial success of KSF and NSF phosphors has prompted a great number of experimental and theoretical studies on Mn^4+^-doped materials in many industrial and academic research groups worldwide.^1−8^

From a practical viewpoint, one of the requirements imposed by LED manufacturers is the high light output of the phosphor at high operating temperatures of the LED device. For example, the operating temperature may reach 90–110 °C in retrofit LED lamps and 125–150 °C in automotive headlamps.^9^ Therefore, much attention has focused on determining the temperature dependence of light output and investigating the mechanism of thermal quenching in Mn^4+^-doped phosphors.^1,2,10−16^ A factor of considerable importance is the activation energy for thermal quenching, which is usually derived indirectly by fitting the measured temperature dependence of the emission intensity or decay time to a modified Arrhenius equation.^16^ Fundamental research that directly characterizes the activation energy of thermal quenching has received comparatively little attention. A rigorous mechanistic understanding of thermal quenching may potentially assist in the discovery and design of new hosts of commercial importance.

Previous work has established that thermal quenching involves a process with two sequential thermally activated crossovers.^2,10,15,16^ The first crossover, which occurs between ^2^E and ^4^T_2_ excited states, is followed by the second crossover between the ^4^T_2_ excited and the ground ^4^A_2_ states. In contrast, other works suggest that quenching involves a crossover between the F^–^ → Mn^4+^ charge-transfer state and ^4^A_2_ ground state.^17,18^

In the archival literature, the Debye temperature (Θ_D_) of the host lattice has been used to screen hosts, which maximizes the quantum efficiency of Ce^3+^ and Eu^2+^ ion luminescence.^19,20^ It was suggested that the Debye temperature is a useful proxy for structural rigidity, and materials with high Debye temperatures supported high efficiency of luminescence. In this work, we inquire if values of Θ_D_ (or structural rigidity) can predict the quantum efficiency and trends in the thermal quenching behavior of the Mn^4+^ ion luminescence in fluoride hosts. Since structural rigidity is strongly related to the elastic properties, we have computed the elastic properties of the pure host lattices.

With this as the background, we can now define the goal of our work, which is to quantitively and systematically establish a predictive structure-optical property relationship governing thermal stability of the Mn^4+^ ion luminescence in fluoride phosphors. The phosphors chosen for investigations are the red-emitting Mn^4+^-doped NSF, NKSF (NaKSiF_6_), and KSF compounds. To achieve this goal, we use the first-principles ΔSCF-DFT technique to model the geometric structures of the ^2^E and ^4^T_2_ excited states and calculate the optical transition energies between these and the ^4^A_2_ ground state. As a part of this investigation, we also determined the reason for the difference in the Mn^4+^ ZPL intensity in NSF, NKSF, and KSF and developed the fractional particle occupancy scheme, which effectively overcomes the computational convergence challenges encountered in the DFT+*U* and hybrid DFT modeling of the ^4^T_2_ excited state of Mn^4+^ ions in solids. These predictive calculation techniques can be applied to other Mn^4+^-doped systems to provide detailed insights into the thermal quenching mechanism.

## 2 Method of Calculations

First-principles calculations within the DFT framework are performed using the Vienna ab initio simulation package (VASP)^21^ employing the projector-augmented-wave pseudopotentials (PAW)^22^ method. We describe the electronic one-electron wave functions with a plane-wave basis with a cutoff of 520 eV energy. All structures were optimized until the maximum force on each atom was less than 0.01 eV/Å, while the electronic energy minimization was performed with the self-consistent field convergence criterion of 10^–6^ eV. The semicore electrons were explicitly treated for Na (2p^6^ 3s^1^), K (3s^2^ 3p^6^ 4s^1^), Si (3s^2^ 3p^2^), F (2s^2^ 2p^5^), and Mn (3d^5^ 4s^2^). Three representative exchange-correlation functionals were employed: the generalized gradient approximation as proposed by Perdew, Burke, and Ernzerhof (PBE),^23^ the strongly constrained and appropriately normed (SCAN) semilocal functional,^24^ and hybrid functionals HSE06 (Short-range Screened-Coulomb PBE exchange combined with PBE correlation).^25^ This was done to check their ability to reproduce the geometric and electronic structure of neat NSF, NKSF, and KSF. The 4 × 4 × 4, 4 × 5 × 4, and 4 × 4 × 5 k-point meshes based on the Monkhorst–Pack scheme^26^ were used for NSF, NKSF, and KSF, respectively. The modeling of Mn^4+^ defects in the fluoride hosts required a supercell, and the transformation matrixes for the construction of the supercells based on the hosts’ structure, are provided in Table S1 of the Supporting Information (SI).^27^ One single k point Γ (Brillouin zone center) was adopted for sampling the Brillouin zone in the total energy and relaxation calculations of the constructed supercell. The geometric structure relaxations of the Mn^4+^-doped systems were calculated using the SCAN, while the electronic structure and optical transitions were calculated using the HSE06 hybrid functional.

**Figure 1:**
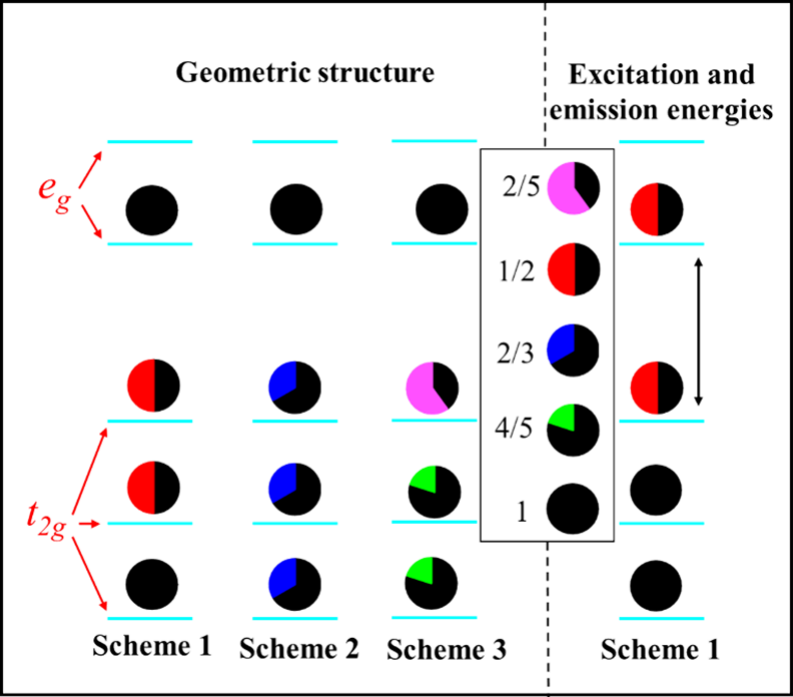
Schematic diagrams of fractional particle occupancy schemes employed to determine the geometric structure of the ^4^T_2_ excited state and to estimate the ^4^A_2_-^4^T_2_ excitation and emission energies, respectively, of Mn^4+^ in an octahedral crystal field. Three t_2g_ orbitals and two e_g_ orbitals are shown separately for the sake of clarity.

In this study, the Mn^4+4^A_2_ ground state, the ^2^E (first) excited state, and the ^4^T_2_ spin-quartet excited state in the fluoride hosts were investigated. The ^2^E and ^4^T_2_ excited states were modeled using the standard Δ*S*CF-DFT.^28,29^ The ^2^E state can be interpreted as arising from a spin flip of one t_2g_ electron, while the ^4^T_2_ state corresponds to a transition of the KS orbital from t_2g_ to e_g_, relative to the ^4^A_2_ ground state. There are no challenges in modeling the ^2^E excited state. The ^4^A_2_-^2^E optical transition energy is calculated by the DFT single determinants, which required adjustment by a scaling factor of 1.5, due to the coexistence of spin components with a value of 1/2 in both the ^2^E and ^4^A_2_ multiplet states.^30^ However, as previously shown, it has been challenging to achieve calculation convergence in modeling the ^4^T_2_ excited state when employing the approximation of the single electronic configuration t2 2ge1 g with the hybrid DFT method. This is due to the significant mixing between the occupied and unoccupied 3d KS orbitals of t_2g_ as found in KSF.^31^ For the interested reader, detailed information about this challenge is provided in Note S1 of the SI.^27^ Herein, we propose using a fractional particle occupancy scheme to effectively address the calculation convergence challenges. Three sets of fractional particle occupancy schemes are used to characterize the geometric structure of the ^4^T_2_ state for reproducing the correct and accurate equilibrium structure along with the optical transition energies. The distribution of two t_2g_ electrons among the three t_2g_ KS orbitals correspond to [ξ ^1^ η^0.5^ ζ^0.5^ θ^1^], [ξ^2/3^η^2/3^ζ^2/3^θ^1^], and [ξ^4/5^η^4/5^ζ^2/5^θ^1^] electron configurations in the Schemes 1, 2, and 3, respectively, as illustrated in Figure 1. Here, ξ, η, and ζ are the components of t_2g_ orbitals while θ and ε are the components of e_g_ orbitals. The Slater’s transition-state method,^32,33^ corresponding to [ξ ^1^ η^1^ ζ^0.5^ θ^0.5^] electronic configuration, was used for the calculation of the excitation and emission energies of the ^4^T_2_-^4^A_2_ optical transition (see the right panel of Figure 1). This is done by the direct use of the eigenvalues of Kohn–Sham orbitals between the lowest half-occupied e_g_ and the highest half-occupied t_2g_ KS orbitals at the equilibrium geometric structures of the ^4^A_2_ and ^4^T_2_ states, respectively. Furthermore, applying the Franck–Condon principle,^33^ the difference in the total energy between the ground and excited state at their respective equilibrium geometric structures was used to determine the zero-phonon line (ZPL) energy of the ^4^T_2_ state.

## 3 Results and Discussion

The KSF, NKSF, and NSF compounds crystallize in the cubic structure with the space group *Fm3̅m* (# 225),^34^ in the orthorhombic structure with the space group *Pnma* (# 62)^35^ and in the trigonal structure with the space group *P3̅m1* (# 164),^36^ respectively. In all structures, the Si^4+^ ions are located at the center of the [SiF_6_]^2–^ octahedron. The Si^4+^site symmetry in KSF and NKSF are *O*_*h*_ and *Ci*, respectively, whereas NSF has two inequivalent Si^4+^ sites with the site symmetry of *D*_3_ and *C*_3_. It is important to choose a suitable exchange-correlation functional in the DFT calculation to reproduce reliably the geometric and electronic structure of a considered system. From the agreement between the computational and experimental lattice parameters, the estimation abilities of the five functionals for the KSF crystal structure is HSE06 ∼ PBE0 > SCAN > PBE > LDA.^31^ To further check the validity of this ranking, the geometric structures of the fluoride hosts with different functionals were evaluated. The calculated structural data are summarized in Table S2 of the SI.^27^ Comparison of the calculated and experimental lattice constants, internal anion position, the [SiF_6_] octahedron volume, and the Si^4+^–F^–^ bond lengths showed that (i) the PBE-calculated results tend to overestimate while the SCAN slightly underestimate the lattice constants with respect to the experimental values; the HSE06-calculated lattice constant is in better agreement with the experimental lattice parameters, and (ii) both HSE06 and SCAN functionals demonstrated better qualitative agreement with the experimental Si^4+^-F^–^ bond length. Notably, the calculated results show good consistency with our earlier studies^31,37−39^ for KSF, although some minor differences arise from variations in the first-principles computational parameters, such as atom pseudopotentials and cutoff energy. Since application of the hybrid functional calculation is time-consuming and considering that good convergence accuracy is achieved using the SCAN functional, all further geometry structure optimizations of the Mn^4+^-doped phosphors were carried out exclusively by this method.

After geometry optimization of the considered structures, their electronic properties were analyzed. The calculated band gap energies of pure NSF, NKSF and KSF, together with the experimental band gap energy of KSF, estimated to be near 9 eV by the cross-luminescence high-energy edge method,^40^ are summarized in Table 3S. To the best of our knowledge, the electronic bandgap of NSF and NKSF compounds has not been experimentally determined. It can be seen that the PBE and SCAN calculations tend to underestimate the experimental value of the band gap of KSF. We wish to point out that previous analysis of DFT calculations has clearly demonstrated that hybrid functionals possess superior predictive capabilities for bandgap estimation, as they consistently show better agreement with the experimental electronic properties of solids.^41,42^ We also performed static HSE06 calculations with the fixed structure obtained from the SCAN functional. The static HSE06/SCAN method yields better experimental-computational agreement, closely matching the results obtained with HSE06, albeit with a slight deviation. A static HSE06 hybrid functional calculation with the SCAN-optimized structure was unable to accurately describe the electronic properties of the pure compounds. This observation suggests that the structural data derived from SCAN calculations can be deemed to be reliable for electronic structure calculations using the HSE06 hybrid functional.

### 3.1 Structural Stability

Practical phosphor host materials should be structurally stable and should not undergo structural phase transitions under the operating conditions of the device. The structural stability of crystalline solids is related to their elastic properties, which are described by the set of elastic constants. The elastic constants of NSF, NKSF, and KSF, calculated using a stress–strain method,^43^ are summarized in Table S4. For mechanical stability, certain criteria of unstressed crystalline structures must be satisfied.^44−46^ The Born stability criteria for elastic stability for all crystal classes are provided in Note S2 of the SI. The criteria for meeting the mechanical stability and the eigenvalues of the stiffness matrix stability for the fluoride hosts are summarized in Tables S5 and S6, respectively. The data of KSF and NSF satisfy both the elastic stability criteria and the eigenvalues of the stiffness matrix stability. However, the required stability conditions are not met in the NKSF system: (i) the elastic stability criteria of 3 and 4 and (ii) the eigenvalues of the stiffness matrix stability criteria of 1 and 2. Accordingly, the KSF and NSF systems are mechanically stable, whereas NKSF is mechanically unstable. This lattice instability may be responsible for the low thermal quenching temperature of Mn^4+^ luminescence in this host, which is discussed later in the paper.

The elastic constants can be used to calculate various elastic properties of the host fluorides.^47^Table S7 presents the elastic parameters calculated using the Voigt–Reuss–Hill (VRH) method. The NKSF system fails to meet the stability condition, resulting in some negative elastic parameters, which is further indicative of its structural instability. This instability leads to an incorrect description of the elastic properties, and consequently, to incorrect values of wave velocities and Debye temperature. Therefore, the elastic properties of NKSF are not considered further.

Table S8 lists the various elastic parameters of the two mechanically stable systems, KSF and NSF. The calculated bulk modulus *B* (GPa) and Debye temperatures Θ_D_ (K) are summarized in Table 1, along with the results obtained in previous studies.^38,39^ Our results are in good agreement with those of the previous studies, although slight differences exist due to differences in calculation methods and parameters, i.e., atom pseudopotentials and cutoff energy. As stated in the Introduction, values of the Debye temperature have been used as predictors of structural rigidity. We investigated if the elastic properties of KSF and NSF hosts affect the quantum efficiency, the Stokes shift in the ^4^A_2_-^4^T_2_ transition, the activation energy for thermal quenching, and the (experimental) quenching temperature *T*_1/2_ of the Mn^4+^ luminescence in these fluoride compounds. This will be discussed in the last section of this paper.

**Table 1:** Calculated Bulk Modulus *B* (GPa) and Debye Temperatures Θ_D_ (K) for NSF and KSF^c^

	*B*		Θ_D_	
system	this work^a^	other works	this work	other works
NSF	41.53		325	
KSF	29.17	16.93^b^	293	280 ^a^, 298 ^b^

a Ref (38).

b Ref (39).

c Note: The averages of the calculated *B* by the Voigt–Reuss–Hill (VRH) method are given here.

### 3.2 Structural and Optical Properties of the Mn^4+2^E and ^4^T_2_ Excited States

We initially focused on obtaining the equilibrium geometric structures of the ^4^T_2_ excited state, employing three sets of fractional particle occupancy schemes tailored to the computational convergence issues outlined in the Computational Methodology section. We selected KSF:Mn^4+^ as the prototype system to determine the effectiveness of our proposed fractional particle occupancy schemes in reliably reproducing the equilibrium geometric structure of the ^4^T_2_ state and thus the associated optical transition energies. Table S10 provides a comparison between the calculated and experimental optical transition energies. It shows that the ^4^T_2_ geometric structure optimized by using the third fractional particle occupancy scheme (Scheme 3) is more accurate and reliable. More detailed information about the superiority of Scheme 3 can be found in Note S3 of the SI. Consequently, Scheme 3 is our preferred choice for the next step, which is the geometry optimization of the ^4^T_2_ state.

The equilibrium geometric structure of Mn^4+^-doped NSF, NKSF, and KSF in both ground and excited states was successfully calculated. Notably, since Si^4+^ ions in NSF have two inequivalent cation sites with *D*_3_ and *C*_3_ symmetries, the more favorable site for Mn^4+^ substitution was identified to be *C*_3_, as shown in Table S11. The results of the calculated Mn^4+^-F^–^ bond length, volume (*V)*, distortion index (*D*), and bond angle variation (σ^2^) of the [MnF_6_]^2–^ octahedron are presented in Table S12. The *D* and σ^2^ of the [MnF_6_]^2–^ octahedron, which are the indices used to characterize the variation of bond length and angles with respect to the ideal octahedron, are determined using the definitions of Baur^48^ and Robenson–Gibbs–Ribbe.^49^

The calculated relative total electronic energies, as shown in Table S11, indicate that the *C*_3_ site is energetically more favorable for Mn^4+^ substitution than the D_3_ site by 0.12 eV for NSF. Therefore, geometric structure relaxations, electronic structure, and optical transition calculations were performed based on Mn^4+^ in the [SiF_6_]^2–^ with *C*_3_ symmetry for NSF. The luminescence properties of Mn^4+^ are strongly influenced by its symmetry and local environment. To the best of our knowledge, the most energetically favorable substitution site for Mn^4+^ in NSF has not been experimentally confirmed. However, experimental photoluminescence spectra show the zero-phonon line (ZPL) intensity of the ^2^E state in NSF:Mn^4+^ (see Figure S1) is strong, which is attributed to the large distortion index of the [MnF_6_] octahedron. An increase in the bond angle variance and distortion index in the ^2^E state results in greater ZPL intensity. The site with *C*_*3*_ symmetry has a bond angle variance and distortion index that are about an order of magnitude larger than those of the *D*_*3*_ symmetry site. Our calculated distortion index (*D*), bond angle variation (σ^2^), and relative total electronic energies for the [MnF_6_]^2–^ octahedron in the ^2^E state, along with experimental photoluminescence spectra, confirm this observation. The antisite defect where the K and Na cations are interchanged is possible in NKSF. Since cation interchange is not possible in KSF and NSF (due to the large difference in the ionic radii and charge), they are considered to be nominally chemically pure and stoichiometric (defect-free).

During the geometry optimization, the structure has a natural tendency to move toward a lower energy configuration, resulting in a descent of the site symmetry. We find that the site symmetry is distorted from *O*_*h*_ to its subgroup *D*_2*h*_ point symmetry in KSF:Mn^4+^, while the site symmetry descends from *C*_3_ in the NSF:Mn^4+^ and *C*_*i*_ in NKSF:Mn^4+^, to the lowest symmetry, C_1._ These results reveal that the substitution of Mn^4+^ leads to a reduction in symmetry and our calculations are consistent with other studies dealing with similar phenomena.^50,51^ Since the ionic radius of the 6-fold coordinated Mn^4+^ is slightly larger than that of Si^4+^,^52^ the Mn^4+^–F^–^ bond-length is expected to be longer than the Si^4+^–F^–^ bond length in pure NSF, NKSF, and KSF. Generally, the average Mn^4+^–F^–^ bond lengths in the ^4^A_2_ ground state show an increase of approximately 0.1 Å compared to the nondoped systems. To the best of our knowledge, only the Mn^4+^–F^–^ bond lengths in KSF:Mn^4+^ have been experimentally estimated. Our calculated result of 1.808987 Å is in good agreement with the experimental value of 1.807 Å.^53^

**Figure 2:**
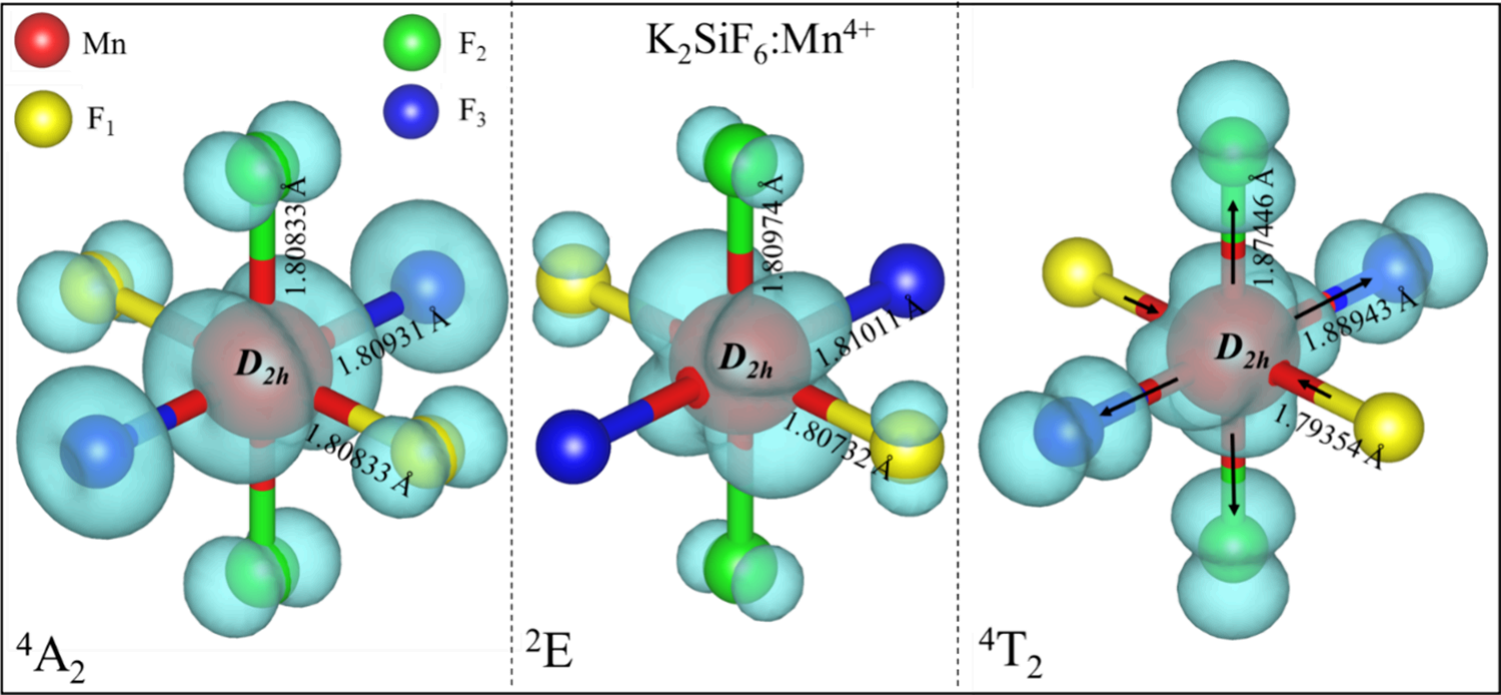
Schematic representation of the local coordination environments of Mn^4+^ impurity ions with charge density distribution of the highest occupied orbitals in the ^4^A_2_ ground state, the ^2^E and ^4^T_2_ excited states of KSF:Mn^4+^. Drawn with VESTA.^56^

In all cases, the Mn^4+^–F^–^ bond lengths in the ^2^E excited state differ very slightly from those in the ^4^A_2_ ground state (by less than 0.003 Å). This minor change is anticipated since the excited ^2^E state involves a spin flip of a t_2g_ electron without any change in the orbital. On the other hand, the ^4^T_2_ excited state undergoes an orbital transition, from t_2g_ to e_g_. The ^4^T_2_ structure undergoes a significant Jahn–Teller distortion where the Mn^4+^–F^–.^ bonds of the [MnF_6_]^2–^ octahedron undergo compression and elongation relative to the ground state. The presence of a strong Jahn–Teller distortion in the ^4^T_2_ states has been observed in previous studies on compounds doped with 3d^3^ ions.^54,55^ To visualize these changes, the local coordination environment of KSF:Mn^4+^ along with the charge density distribution of the highest occupied orbital of the majority spin for the ^4^A_2_ and ^4^T_2_ states and the minority spin for the ^2^E state are plotted in Figure 2. It can be seen that the Mn^4+^–3d orbitals contain substantial contributions from the 2p orbitals of the F^–^ ligands in each state.

Experimental photoluminescence data reveals a significant difference in the ^2^E → ^4^A_2_ ZPL intensity among Mn^4+^-doped NSF, NKSF, and KSF.^57^ We attribute this difference to the local coordination environment of the [SiF_6_]^2–^ octahedra in the pure hosts. Given that the ZPL intensity is linked to the local coordination environment of [MnF_6_]^2–^ in the ^2^E state, and there is a significant difference between the local coordination environment of [MnF_6_]^2–^ and [SiF_6_]^2–^, the observed difference in the ZPL intensity can be explained by considering the local coordination environment of the [MnF_6_]^2–^ cluster in the Mn^4+2^E electronic state, which forms after the substitution of Si^4+^ by Mn^4+^.

The experimental photoluminescence spectra of Mn^4+^-doped NSF, NKSF, and KSF^57^ along with the calculated distortion parameters *D* and σ^2^ of the [MnF_6_]^2–^ octahedron in the ^2^E state are shown in Figure S1 of the SI. The distortion index and bond angle are key parameters characterizing the ZPL intensity. The *D* and σ^2^ of the [MnF_6_]^2–^ moiety in its ^2^E electronic state are the largest in NKSF:Mn^4+^ and smallest in KSF:Mn^4+^. Experimentally, the strongest ZPL intensity is indeed observed in NKSF and the weakest (near zero) in KSF (see Figure S1 of the SI). Generally, the ZPL intensity increases with increasing *D* and σ^2^ values of the [MnF_6_]^2–^ octahedral moiety in the ^2^E state. Therefore, we observe a good agreement between the theoretical calculations and experimental data.

**Figure 3:**
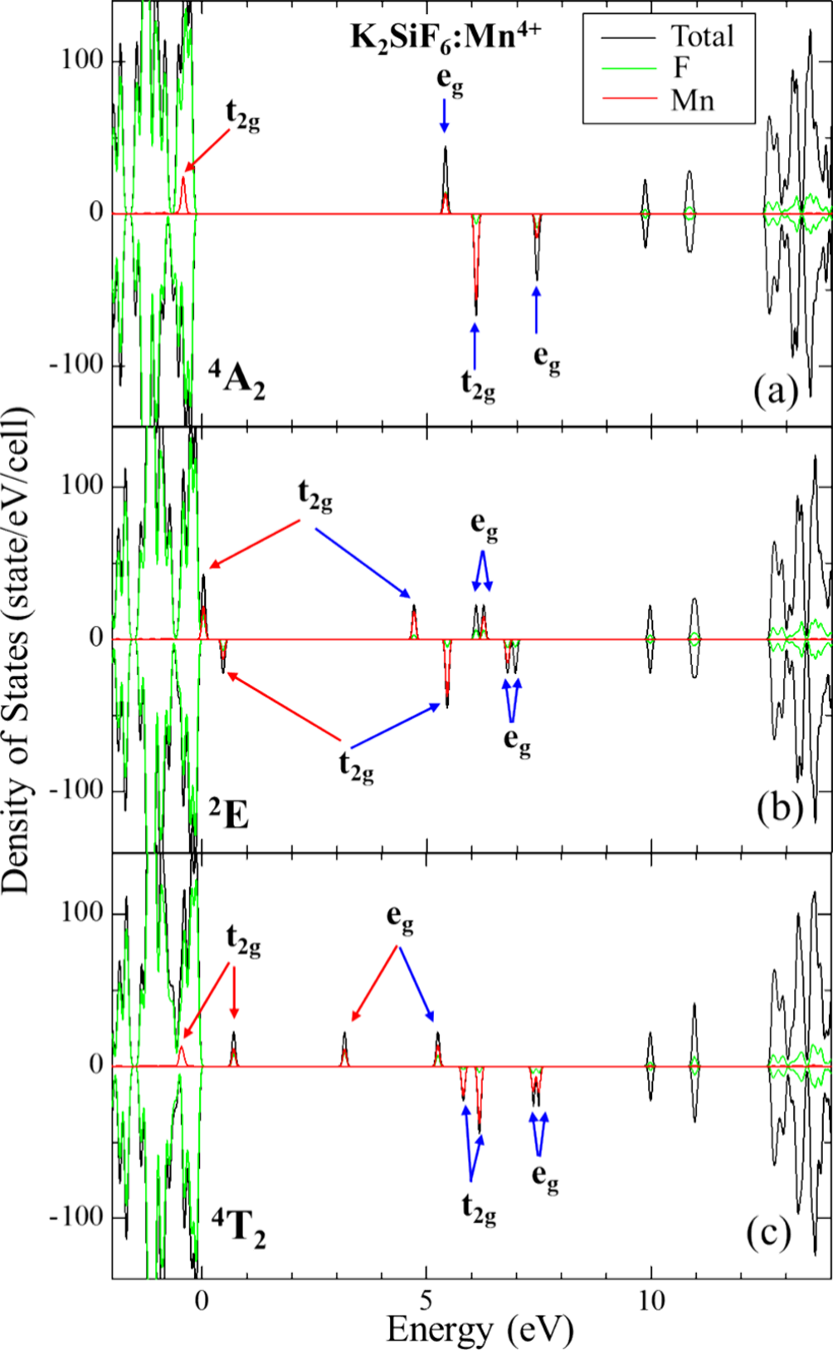
Calculated electronic density of states of the ^4^A_2_ ground state (a), the ^2^E (b), and ^4^T_2_ (c) excited states for KSF:Mn^4+^ using HSE06. The red and blue arrows indicate the occupied and unoccupied e_g_ and t_2g_ KS orbitals, respectively. For the ^4^T_2_ state, the [ξ^0.8^η^0.8^ζ^0.4^θ^1^] single electronic configuration was used to calculate the DOS based on the geometric structure optimized by the third fractional particle occupancy (Scheme 3).

After geometric optimization of the considered states, their electronic structures were calculated. The electronic density of states (DOS) for both the ground and the excited states of Mn^4+^ doped NSF, NKSF, and KSF were obtained using the HSE06 calculations. Since the DOS diagrams showed no significant differences between the doped fluorides, only the DOS diagrams for KSF:Mn^4+^ are presented in Figure 3. The DOS diagrams for Mn^4+^-doped NKSF and NSF are shown in Figure.S2. In the octahedral environment, the 3d orbitals of Mn^4+^ split into the lower 3-fold degenerate t_2g_ and the higher 2-fold degenerate e_g_ orbitals in each spin direction. The DOS diagrams for the ^4^A_2_ ground state reveal that the substitution of Mn^4+^ leads to the appearance of four new states within the bandgap (two states for each spin-up and spin-down direction). The Mn^4+^ 3d states hybridize with the ligand F 2*p* states in all triply and doubly degenerated t_2g_ and e_g_ orbitals. In both excited states, the site symmetry of the Mn^4+^ ions was found to be *D*_2*h.*_. The t_2g_ KS orbitals split into three singlets *B*_1_, *B*_2_, and *B*_3_ orbitals, while the e_g_ KS orbitals transform into doubly degenerate *A*_1_ orbitals.^58^ The two singlets *B*_1_ and *B*_2_ orbitals are very close together in the ^2^E and ^4^T_2_ states. To understand the J-T distortion for each excited state, we examined the energy gap between doubly degenerate *A*_1_ orbitals of the e_g_ KS orbitals in the ^2^E and ^4^T_2_ states with spin up. The comparison of the energy gap between doubly degenerate *A*_1_ orbitals of the e_g_ KS orbitals in the ^2^E and ^4^T_2_ states reveals that the J-T distortion in the ^4^T_2_ state is greater than that in the ^2^E state, which is consistent with the geometric structure results. The ^2^E excited state undergoes a small Jahn–Teller distortion, whereas the ^4^T_2_ state shows greater distortions in the nearest environment, resulting in a stronger splitting of the t_2g_ orbitals.^54,55^

The energies of the optical transition between the ^4^A_2_ ground and ^2^E and ^4^T_2_ excited states were calculated by using HSE06. The experimental-theoretical comparison of the optical transition energies is provided in Table 2. We observe good agreement between the DFT- Δ*S*CF technique and the experimental optical transition energies for all phosphors. The emission energies of the ^4^A_2_-^2^E optical transition calculated by HSE06 are slightly lower than the experimental values, with differences of less than 0.08 eV (Table 2). Due to minor changes in the local coordination environments around the Mn^4+^ ion in the ^4^A_2_ and ^2^E states, the difference between the calculated ^2^E → ^4^A_2_ (emission) and ^4^A_2_ → ^2^E (excitation) transitions is very small, less than 0.01 eV. Although the calculated ^4^A_2_ → ^4^T_2_ excitation energies are slightly higher than the experimental values, the trends are qualitatively reproduced by the current DFT calculations. Specifically, the excitation energy decreases in the sequence NKSF - KSF - NSF, which is the order of increasing Mn^4+^–F^–^ bond lengths in the ^4^A_2_ state. Notably, the experimental ZPL energy of the ^4^T_2_ state in KSF:Mn^4+^ is well reproduced theoretically.^59^ The experimental values of the ZPL energy of the ^4^T_2_ state for the other two phosphors are currently not available.

**Table 2:** Comparison of the Calculated and Experimental Excitation, Emission, and ZPL and Stokes Shift Energies of the Optical Transitions between the Ground State ^4^A_2_ and the Excited States ^2^E and ^4^T_2_ of Mn^4+^ Dopants in NSF, NKSF, and KSF (All in eV)

		NSF: Mn^4+^		NKSF: Mn^4+^		KSF: Mn^4+^	
transition type	state	calc.	exp.^a^	calc.	exp.^b^	calc.	exp.^c^
excitation	^2^E	1.9301		1.9571		1.9216	2.0–2.1
	^4^T_2_	2.7600	2.72	2.8223	2.6953	2.7990	∼2.7
emission	^2^E	1.9221		1.9487		1.9143	1.968
	^4^T_2_	2.3799		2.4532		2.4579	
ZPL	^2^E	1.9268	2.0126	1.9548	1.9968	1.9188	1.9982
	^4^T_2_	2.6400		2.7037		2.6893	2.676

a Ref (2).

b Ref (57).

c Ref (59).

**Figure 4:**
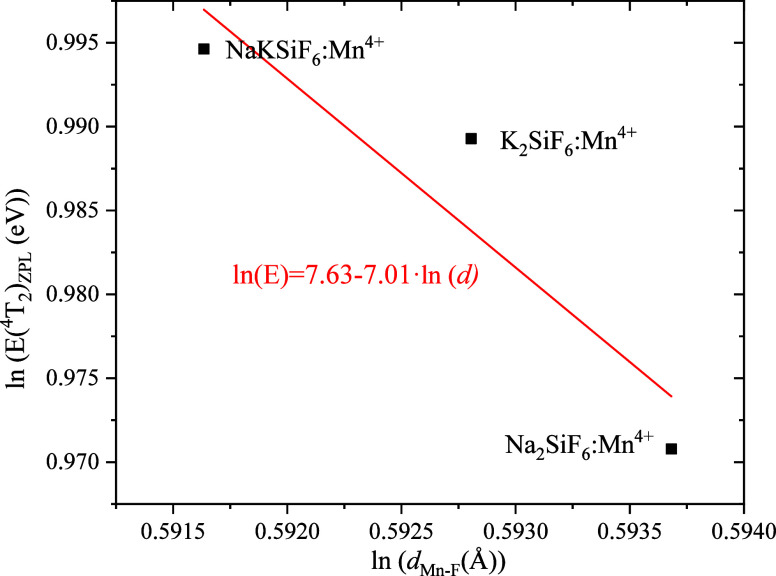
Calculated ZPL energies of ^4^T_2_ state for Mn^4+^ dopants in NSF, NKSF, and KSF systems, as a function of Mn^4+^–F^–^ bond length. The *d*_Mn–F_ bond length is the average Mn^4+^–F^–^ bond lengths in the equilibrium geometric structure of the ^4^A_2_ ground state.

The dependence of the ZPL energies of the ^4^T_2_ state as a function of the average Mn^4+^–F^–^ bond lengths in the equilibrium geometric structure of the ^4^A_2_ ground state is plotted in Figure 4. An increase in the interionic distance is accompanied by a decrease in the energy of the ZPL. The dependence of the ZPL energies of ^4^T_2_ on the Mn^4+^-ligand distance is assumed to vary as E(^4^T_2_) _ZPL_ = *kd*^–n^. In the framework of the point charge model of crystal field, *n* = 5. For real systems, *n* ≥ 5.^60^ The variation of the ZPL energies for the ^4^T_2_ state is fitted to the equation: *E*(^4^T_2_) _ZPL_ = *kd*^–*n*^. The E(^4^T_2_) _ZPL_ emission energy depends on *d*_Mn–F_: ln(*E*)=7.63–7.01·ln(*d*). This theoretical expectation is fully confirmed by the DFT-evaluated equilibrium geometric structures of the states with accurate optical transition energies and impurity-ligand distance within [MnF_6_]^2–^ octahedron clusters as well. Importantly, our accurate prediction of optical transition energies will be key in providing an understanding of the thermal quenching in these phosphor systems.

### 3.3 Thermal Quenching Mechanism

**Figure 5:**
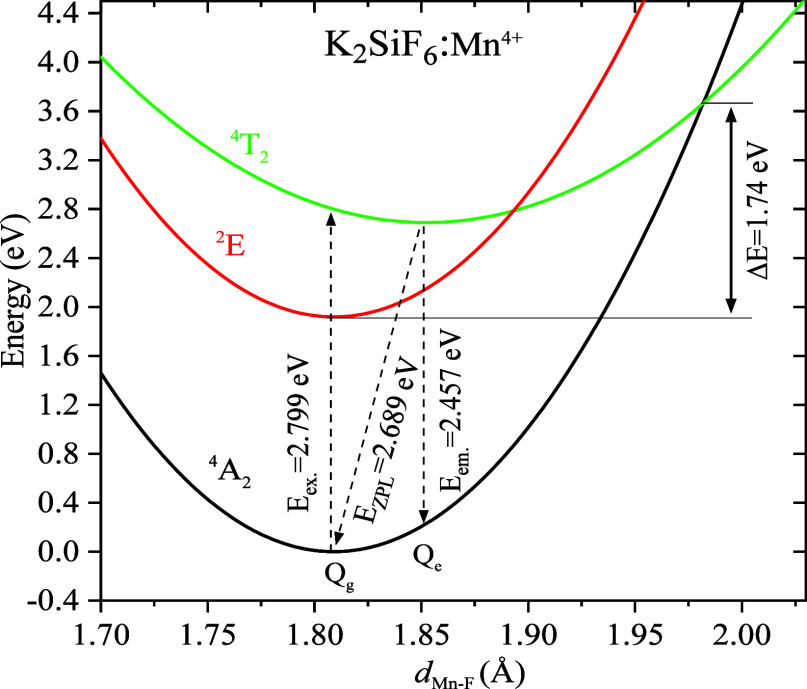
Schematic depiction of the calculated configuration coordinate diagram of the Mn^4+^ ion impurity in K_2_SiF_6_. *Q*_g_ and *Q*_e_ are the average over the six Mn^4+^–F^–^ bond lengths of the equilibrium geometric structure of ^4^A_2_ and ^4^T_2_ states, respectively. *E*_ex._, *E*_em._, *E*_ZPL_, and Δ*E* represent the calculated excitation, emission and ZPL energies of the optical transition ^4^A_2_-^4^T_2_, and the activation energy for thermal quenching, respectively.

Figure 5 shows the one-dimensional configuration coordinate diagram of the Mn^4+^ impurity in K_2_SiF_6_, which was constructed based on the optimized equilibrium structures and the optical transition energies. The diagram was constructed by applying a one-dimensional harmonic approximation for the potential surfaces along with the equilibrium structure points of the ^4^A_2_, ^2^E, and ^4^T_2_ states and with the calculated excitation, emission, and ZPL energies of the ^4^A_2_-^2^E and ^4^A_2_-^4^T_2_ optical transitions. The calculated average Mn^4+^-F^–^ bond lengths of the [MnF_6_]^2–^ complex in the ground ^4^A_2_ and excited ^4^T_2_ states are indicated by the *Q*_*g*_ and *Q*_*e*_ points along the horizontal axis, respectively. These correspond to the lowest energy potential surfaces of the ground ^4^A_2_ and excited ^4^T_2_ states.

In the crossover model, thermal quenching of the Mn^4+^ emission in fluorides corresponds with the energy needed to reach the crossover between the potential surfaces of the ground ^4^A_2_ and excited ^4^T_2_ states.^10,14,15^ The activation energies (Δ*E*) for thermal quenching of Mn^4+^-doped NSF, NKSF, and KSF were estimated to be 1.566, 1.687, and 1.7445 eV, respectively. In Table 3, we assembled the calculated spectral parameters and the experimental values of the onset temperature of thermal quenching (*T*_onset_) and the temperature at which the emission intensity has fallen to 50% of its initial value (*T*_1/2_) for the NSF and KSF phosphors.

**Table 3:** Computed Spectral Parameters for Mn^4+^-Doped NSF, NKSF, and KSF^a^

system	^4^A_2_→ ^4^T_2_ (ex.) (eV)	^4^T_2_ → ^4^A_2_ (em.) (eV)	Stokes shift (eV)	Δ*E* (eV)	^4^T_2_ (ZPL) (eV)	*T*_onset_ (K)	*T*_1/2_ (K)	θ_D_ (K)
NSF	2.76	2.38	0.38	1.56	2.64	400	430	325.1
NKSF	2.82	2.45	0.37	1.68	2.70			
KSF	2.79	2.45	0.34	1.74	2.68	450	500	292.5

a The experimental *T*_onset_ and *T*_1/2_ values for NSF and KSF are from refs (2) and (16), respectively

In fluoride compounds, the key parameter determining the activation energy, *T*_1/2_, and *T*_onset_ is the energy position of the ^4^T_2_ state. Generally, the higher the energy of the ^4^T_2_ state, the higher are the values of these parameters.^2,10,16^ The calculated energy of the ^4^T_2_ state in KSF is 0.05 eV higher than that of the NSF phosphor. This trend in the activation energy is consistent with the experimental data: the activation energy for thermal quenching is less for NSF than that in KSF.^2^ In consequence, Δ*E*, *T*_onset_, and *T*_1/2_ are higher for KSF.

The activation energy for thermal quenching of NKSF:Mn^4+^ is higher than that for the NSF:Mn^4+^ phosphor. Experimentally, thermal quenching of Mn^4+^ luminescence in NKSF occurs at temperatures much lower than that in NSF.^13^ This reveals that thermal quenching in NKSF does not occur via the ^2^E → ^4^T_2_ → ^4^A_2_ crossover mechanism. Therefore, we postulate that thermal quenching in NKSF is due to other nonradiative decay pathways such as energy transfer to lattice defects. We have previously shown that the elastic properties of NKSF are indicative of a mechanically unstable system. We suggest that this instability is connected to the lower thermal quenching temperature of the embedded Mn^4+^. This inherent instability is also reflected in the decay kinetics of luminescence, which reveals two distinct Mn^4+^ sites in NKSF.^61^ This does not agree with the reported crystal structure, which indicates a single site for Mn^4+^ substitution.

Our computation works show that apart from NKSF, thermal quenching of Mn^4+^ luminescence in fluoride systems occurs by the ^2^E → ^4^T_2_ → ^4^A_2_ crossover mechanism. It leads to the conclusion that the dependence of activation energy on the energy of the ^4^T_2_ level is a robust electronic structure–property relationship in fluorides.

The data in Table 3 also serve to illustrate the influence of Stokes shift on thermal quenching of luminescence. Even though the energy of the ^4^T_2_ state is higher in NKSF, the activation energy for thermal quenching is lower than is calculated for KSF. However, the Stokes shift in the ^4^A_2_-^4^T_2_ transition is smaller in KSF. Due to the smaller Stokes shift, the ^4^T_2_ → ^4^A_2_ crossover occurs at a higher energy in KSF than in NKSF. Therefore, the difference in the activation energy of KSF and NSF reflects not only the higher energy of the ^4^T_2_ state but also the smaller Stokes shift of the ^4^T_2_ -^4^A_2_ transition in the KSF phosphor. In a single configuration model, a small Stokes shift of the ^4^T_2_ state will increase the activation energy for thermal quenching. Although, a small Stokes shift may also imply a greater probability of energy transfer via the ^4^T_2_ level, thermal quenching in KSF and NSF occurs via the ^2^E → ^4^T_2_ → ^4^A_2_ crossover process. We conclude that such calculations can help verify the mechanism of thermal quenching and thus more broadly assist in the smart search for novel Mn^4+^-doped phosphors with high thermal stability.

We assume that antisite defects (disorder between K and Na) contribute to nonradiative recombination processes in NKSF. This type of antisite defect does not occur in K_2_SiF_6_ (KSF) and Na_2_SiF_6_ (NSF). The ^2^E → ^4^T_2_ → ^4^A_2_ crossover process does not contribute to the observed behavior of NKSF since our calculation suggests a higher activation energy for the crossover process in NKSF. Experimentally, the thermal quenching in NSF occurs at higher temperatures. A more appropriate description of the quenching process in NSKF is ^2^E → Defect → ^4^A_2_, with lower activation energy than the ^2^E → ^4^T_2_ → ^4^A_2_ crossover process.

To seek the relationship between the elastic properties of the hosts and the thermal quenching parameters, we have compiled in Table 4 the calculated activation energy for thermal quenching Δ*E*, the bulk modulus *B* and Debye temperatures Θ_D_ along with the experimental quenching temperature (*T*_1/2_), of Mn^4+^-doped NSF and KSF. Materials with lower Θ_D_ tend to increase the probability of nonradiative decay, leading to lower quantum efficiency and *T*_1/2_.^19,20^ Despite the difference in the Debye temperature: (1) the quantum efficiency of commercial NSF and KSF is similar, and (2) the *T*_1/2_ of NSF:Mn^4+2^ is lower than that in KSF:Mn^4+^.^16^ Therefore, structural rigidity, as defined by the value of the Debye temperature, is not a reliable parameter for fluoride host selection with targeted optical and thermal properties.^62,63^ Note from the data of Table 3 that the Debye temperature (or structural rigidity) also does not account for the trend in the Stokes shift (nonradiative relaxation) in the ^4^A_2_-^4^T_2_ transition. The quantum efficiency of NSF is about 96% of that of KSF. Therefore, despite the higher Debye temperature of NSF, the quantum efficiency is actually a bit lower.

**Table 4:** Calculated Activation Energy for Thermal Quenching Δ*E*, Bulk Modulus *B*, and Debye Temperatures Θ_D_ Together with Experimental Quenching Temperature of *T*_1/2_ of Mn^4+^-Doped A_2_SiF_6_ (A = Na, K)

	Δ*E* (eV)	*B* (GPa)	Θ_D_ (K)	*T*_1/2_ (K)
system	calc.	calc.	calc.	exp.
NSF: Mn^4+^	1.5665	41.53	325	430^a^
KSF: Mn^4+^	1.7445	29.17	293	500^b^

a Ref (2).

b Ref (16).

While the Debye temperature provides certain insights into the vibrational properties of a solid and is related to the lattice’s ability to conduct heat, it does not directly correlate with the thermal stability of luminescence, which depends on more specific factors such as the electronic transitions, defect states, and quenching mechanisms in the luminescent material. Therefore, other factors like activation energy for thermal quenching or the stability of the excited states are often more relevant for assessing luminescence thermal stability.^62,63^

## 4 Conclusions

This systematic research has provided insights into the origin of the electronic properties responsible for thermal quenching of the Mn^4+^ ion luminescence in fluoride hosts. First-principles calculations on Mn^4+^-doped Na_2_SiF_6_ (NSF), NaKSiF_6_ (NKSF), and K_2_SiF_6_ (KSF) were used to explore how host properties and local coordination environments influence the thermal quenching behavior. The geometric structures of the Mn^4+2^E and ^4^T_2_ excited states, together with their optical transition energies relative to ground state ^4^A_2_, were successfully modeled. We find good agreement between the DFT- calculated energies for the excitation, emission, and Stokes shift associated with the optical transitions ^4^A_2_-^2^E and ^4^A_2_-^4^T_2_ and the experimental data. Thermal quenching in Mn^4+^ doped KSF and NSF occurs by the ^2^E → ^4^T_2_ → ^4^A_2_ crossover mechanism. In contrast, thermal quenching in NKSF is due to other nonradiative decay pathways such as energy transfer to lattice defects. We find from the analysis of the elastic properties that NKSF is mechanically unstable. We suggest that this property of the host limits the luminescence efficiency of embedded Mn^4+^.

Through the fractional particle occupancy scheme developed in this study, the convergence challenges, employing the approximation of the single electronic configuration t2 2ge1 g, were effectively addressed in modeling of the ^4^T_2_ excited state for Mn^4+^ dopant in solids. The general applicability of the scheme, which effectively overcomes the computational convergence challenges present in the DFT calculations, opens a novel avenue for high-throughput search for new phosphors. The work provides a new perspective for better and deeper understanding of the thermal quenching mechanism in Mn^4+^-doped fluorides and can be used to design and optimize Mn^4+^-doped fluoride phosphors with higher quenching temperatures and enhanced thermal stability.

## Supporting Information Available

Additional computational details and calculated results (PDF)
