# Supplementary material for: Thermal Quenching Mechanism of Mn4+ in Na2SiF6, NaKSiF6, and K2SiF6 Phosphors: Insights from the First-Principles Analysis
Source: Inorg Chem. 2024 Oct 21;63(44):21212–21. doi: 10.1021/acs.inorgchem.4c03589 (PMC11539052; doi:10.1021/acs.inorgchem.4c03589)
Supplement: Supplementary file 1 — ic4c03589_si_001.pdf [file ic4c03589_si_001.pdf]

Supporting Information

for "Thermal Quenching Mechanism of Mn^{4+} In Na_2SiF_6, $NaKSiF_6$ and K_2SiF_6 phosphors: Insights from the first-principles analysis"

Mekhrdod S. Kurboniyon[1,2,3], Alok M. Srivastava[4*], Bibo Lou[1], Dilshod D. Nematov[5], Amondulloi Burhonzoda[5], Tomoyuki Yamamoto[3], Chong-Geng Ma[1,2*], Mikhail G. Brik[1,6,7,8*]

[1] *School of Optoelectronic Engineering & CQUPT-BUL Innovation Institute, Chongqing University of Posts and Telecommunications, Chongqing 400065, China*

[2] *National Academy of Sciences of Tajikistan, Dushanbe 734025, Tajikistan*

[3] *Kagami Memorial Research Institute for Materials Science and Technology, Waseda University, Tokyo 169-0051, Japan*

[4] *Current Lighting Solutions LLC, 1099 Ivanhoe Road, Cleveland, OH 44110, USA*

[5] *Physical–Technical Institute, National Academy of Sciences of Tajikistan, Dushanbe 734063, Tajikistan*

[6] *Centre of Excellence for Photoconversion, Vinča Institute of Nuclear Sciences - National Institute of the Repub-lic of Serbia, University of Belgrade, Belgrade, Serbia*

[7] *Institute of Physics, University of Tartu, W. Ostwald Str. 1, Tartu 50411, Estonia*

[8] *Academy of Romanian Scientists, 3 Ilfov, 050044, Bucharest, Romania*

[*] Corresponding author, *E*-mail: srivastaam@outlook.com (A.M Srivastava)

[*] Corresponding author, *E*-mail: cgma.ustc@gmail.com (C.-G. Ma)

[*] Corresponding author, *E*-mail: mikhail.brik@ut.ee (M.G. Brik)

Table S1. The transformation matrixes for the construction of the supercells based on the fluoride hosts' structure: Na_2SiF_6 (NSF), $NaKSiF_6$ (NKSF) and K_2SiF_6 (KSF).

System	Matrix
NSF<br>(**P-3m1** (164))	$\begin{pmatrix} 1 & 0 & 0 \\ 0 & 1 & 0 \\ 0 & 0 & 2 \end{pmatrix}$
NKSF<br>(**Pnma** (62))	$\begin{pmatrix} 1 & 1 & 0 \\ -1 & 1 & 0 \\ 0 & 0 & 1 \end{pmatrix}$
KSF<br>(**Fm-3m** (225))	$\begin{pmatrix} 2 & 0 & 0 \\ 0 & 2 & 0 \\ 0 & 0 & 2 \end{pmatrix}$

Note S1.

To be able to compute the optical transitions of ions, as in our case of the Mn^{4+} ion, it is important to obtain a correct and accurate equilibrium geometric structures of the ground and excited states. We first focused on the obtaining the equilibrium geometric structures of the states. In this work, the $^4A_2$ ground and ^2E and $^4T_2$ exited states of the Mn^{4+} ion in an octahedral field is discussed. Computationally, the equilibrium geometric structure of the ^2E excited state of 3d ions did not present any challenges. However, previous investigations have shown that the geometry optimization of the $^4T_2$ excited state in KSF: Mn^{4+} is challenging in hybrid functional calculation. This is because the energy gap between the lowest occupied e_g and the highest unoccupied t_{2g} KS orbitals is narrow and during the geometry optimization, the strong mixing between these two KS orbitals can cause the calculation collapse [S1]. We also encountered a computational breakdown when describing the $^4T_2$ of Cr^{3+} ions in $Ca_3Y_2Ge_3O_{12}$ due to the significant mixing between the constrainedly occupied and unoccupied 3d KS orbitals of Cr^{3+}. Thus, not only the hybrid DFT calculation, but also the PBE+*U* functional for the geometry optimization of the $^4T_2$ state of $Ca_3Y_2Ge_3O_{12}$: Cr^{3+} with the single electronic configuration t2 2ge1 g failed completely. This is not surprising, given that the significantly narrow t_{2g}-e_g energy gap of Cr^{3+} ions in $Ca_3Y_2Ge_3O_{12}$ intensifies the mixing between the lowest occupied e_g and the highest unoccupied t_{2g} KS orbitals, pushing the calculations towards collapse [S2].

Additionally, we carried out the static HSE06 hybrid electronic structure calculation of the energy level position of t_{2g} and e_g KS orbitals of Mn^{4+} impurity in KSF with the single electronic

configuration t2 2ge1 g based on the fixed equilibrium geometric structures of the $^4A_2$ ground state. We could not achieve convergence even in the static HSE06 electronic structure calculation because the lowest occupied e_g and the highest unoccupied t_{2g} KS orbitals are mixed. Furthermore, we conducted a series of static hybrid calculations with the single electronic configuration t2 2ge1 g based on the fixed equilibrium geometric structures of the $^4A_2$ ground state by varying the amount of HF exchange mixed into the HSE06 functional, from 0 to 25% in 5% increments. With these modified hybrid functionals, we could not achieve convergence even in the static HSE06 electronic structure calculation when the mixed HF exchange is larger than 15%. However, the two t_{2g} electrons can partially infiltrate into the highest empty t_{2g} KS orbitals to avoid the challenge of orbital mixing. Therefore, we have proposed the fractional particle occupancy schemes as a suitable way to reproduce correct and accurate equilibrium geometric structure of the $^4T_2$ state, as well as optical transitions.

Table S2. Comparison of the calculated and experimental structural properties of NSF, NKSF and KSF systems.

System	Parameter	Calc. (Å)			Exp. (Å)
		PBE	SCAN	HSE06	
NSF	a=b (Å)	9.0181	8.7801	8.9192	8.859 [a]
	c (Å)	5.1156	4.9464	5.0555	5.038
	Si(1) (*x*, *y*, *z*)	(0.0, 0.0, 0.0)	(0.0, 0.0, 0.0)	(0.0, 0.0, 0.0)	(0.0, 0.0, 0.0)
	Si(2) (*x*, *y*, *z*)	(0.33333, 0.66667, 0.50558)	(0.3333, 0.66667, 0.50300)	(0.33333, 0.66667, 0.50509)	(0.33330, 0.66670, 0.50600)
	F(1) (*x*, *y*, *z*)	(0.09535, 0.18059, 0.80858)	(0.09842, 0.18271, 0.80415)	(0.09574, 0.17999, 0.80818)	(0.08700, 0.09200, 0.81000)
	F(2) (*x*, *y*, *z*)	(0.44208, 0.59650, 0.69923)	(0.44326, 0.59536, 0.70059)	(0.44222, 0.59733, 0.69855)	(0.44400, 0.40100, 0.70100)

	F(3) (*x*, *y*, *z*)	(0.22676, 0.73969, 0.31199)	(0.22554, 0.74099, 0.30569)	(0.22698, 0.73915, 0.31183)	(0.23000, 0.26000, 0.31000)
	$Si(1)^{4+}$-F^{-}1×6 (Å)	1.71759	1.69484	1.69588	1.67414
	$V([Si(1)F_6]^{2-})$(Å^3)	6.7475	6.4774	6.4936	6.2537
	$Si(2)^{4+}$-F^{-}2×3 (Å)	1.72169	1.69794	1.69797	1.69510
	$Si(2)^{4+}$-F^{-}3×3(Å)	1.72368	1.70047	1.69877	1.68221
	$V([Si(2)F_6]^{2-})$ (Å^3)	6.8158	6.5408	6.5313	6.4166
NKSF	a (Å)	9.5710	9.3271	9.3718	9.3387 [b]
	b (Å)	5.5409	5.3691	5.4434	5.5032
	c (Å)	9.8349	9.5048	9.7973	9.7957
	F(1) (*x*, *y*, *z*)	(0.32689, 0.47076, 0.99879)	(0.32435, 0.47469, 0.99414)	(0.32729, 0.47228, 0.99901)	(0.32510, 0.47230, 0.99120)
	F(2) (*x*, *y*, *z*)	(0.12562, 0.02994, 0.85142)	(0.12829, 0.02545, 0.84767)	(0.12558, 0.02917, 0.85224)	(0.1331, 0.0363, 0.8581)
	F(3) (*x*, *y*, *z*)	(0.11772, 0.25000, 0.06181)	(0.10978, 0.25000, 0.06133)	(0.11650, 0.25000, 0.06028)	(0.1162, 0.25000, 0.05420)
	F(4) (*x*, *y*, *z*)	(0.33775, 0.25000, 0.78398)	(0.34506, 0.25000, 0.78732)	(0.33902, 0.25000, 0.78619)	(0.34120, 0.25000, 0.78820)
	Si^{4+}-F^{-}1×2 (Å)	1.72048	1.69870	1.69475	1.66218[b]
	Si^{4+}-F^{-}2×2 (Å)	1.71238	1.68696	1.69147	1.60517
	Si^{4+}-F^{-}3×1 (Å)	1.70952	1.68720	1.68448	1.6430
	Si^{4+}-F^{-}4×1 (Å)	1.74025	1.71846	1.71555	1.7130
	$V([SiF_6]^{2-})$ (Å^3)	6.7707	6.5008	6.4926	5.9261
KSF	a=b=c (Å)	8.2626	7.9845	8.1628	8.1341[c]

	F (x, y, z)	(0.20829, 0.0, 0.0)	(0.21257, 0.0, 0.0)	(0.20795, 0.0, 0.0)	(0.20692, 0.0, 0.0)
	Si^{4+}-F^{-}×6 (Å)	1.72108	1.69735	1.69754	1.6829[a]
	$V([SiF_6]^{2-})$ (Å^3)	6.7974	6.5201	6.5223	6.3553

[a] Ref. S3

[b] Ref. S4

[c] Ref. S5

Table S3. Calculated band gap of NSF, NKSF and KSF compounds by different exchange-correlation functionals, together with experimentally measured band gap for KSF (all in units of eV).

System	Calc. (Å)				Exp. [a]
	PBE	SCAN	HSE06	HSE06/SCAN	
NSF	6.9808	8.2611	9.4676	9.7774	~ 9
NKSF	6.9889	8.2493	9.4642	9.7260	
KSF	7.1873	8.6160	9.6677	9.9377	

[a] Ref. S6

Table S4. Calculated elastic constants C_{ij} (all in GPa) for the NSF, NKSF and KSF systems by the PBE method.

System	Parameter	Calc.
NSF	C_{11}	68.523
	C_{12}	33.792
	C_{13}	28.884
	C_{14}	2.740
	C_{44}	10.458
	C_{66}	17.366
NKSF	C_{11}	24.162
	C_{12}	49.498

	C_{13}	33.595
	C_{22}	-149.464
	C_{23}	-63.088
	C_{33}	18.253
	C_{44}	-8.062
	C_{55}	17.508
	C_{66}	15.405
KSF	C_{11}	36.102
	C_{12}	25.718
	C_{44}	22.269

Note S2

Mechanical stability requires that the elastic constants should satisfy the following conditions for unstressed crystalline structures [S7]:

$$C_{11} > 0; \quad (1)$$

$$C_{11}C_{22} > (C_{12})^2; \quad (2)$$

$$C_{11}C_{22}C_{33} + 2C_{12}C_{13}C_{23} - C_{11}(C_{23})^2 - C_{22}(C_{13})^2 - C_{33}(C_{12})^2 > 0; \quad (3)$$

$$C_{44} > 0; \quad (4)$$

$$C_{55} > 0; \quad (5)$$

$$C_{66} > 0. \quad (6)$$

In particular, the conditions for the stability of cubic systems can be reduced to a very simple form:

$$C_{11} > 0, C_{11} + 2C_{12} > 0, C_{44} > 0. \quad (7)$$

Table S5. The meeting criteria of the elastic stability for NSF, NKSF and KSF systems.

System	Elastic stability criteria

	1	2	3	4	5	6
NSF	yes	yes	yes	yes	yes	yes
NKSF	yes	yes	**no**	**no**	yes	yes
KSF	yes	yes	yes	yes	yes	yes

Table S6. The meeting criteria of eigenvalues of the stiffness matrix stability for NSF, NKSF and KSF systems.

System	Stiffness matrix criteria					
	1	2	3	4	5	6
NSF	yes	yes	yes	yes	yes	yes
NKSF	**no**	**no**	yes	yes	yes	yes
KSF	yes	yes	yes	yes	yes	yes

Table S7. Calculated elastic properties of NSF, NKSF and KSF systems by Voigt [S8], Reuss [S9] and Hill [S10] methods.

Mechanical properties	System								
	NSF			NKSF			KSF		
	Voigt	Reuss	Hill	Voigt	Reuss	Hill	Voigt	Reuss	Hill
Bulk Modulus B (GPa)	41.850	41.211	41.530	-7.45	26.569	9.560	29.180	29.179	29.179
Young's Modulus E (GPa)	38.89	35.617	37.257	-9.08	104.031	43.215	39.370	25.995	32.778
Shear Modulus G (GPa)	14.450	13.134	13.794	-3.50	61.381	28.941	15.440	9.617	12.528
Poisson's Ratio v	0.350	0.356	0.350	0.30	-0.153	-0.253	0.280	0.352	0.312
P-wave Modulus (GPa)	61.120	58.722	59.992	-12.12	108.411	48.148	49.760	42.002	45.883
Pugh's Ratio (B/G)	2.900	3.138	3.011	2.13	0.433	0.330	1.890	3.034	2.329

Table S8. Calculated elastic properties of NSF and KSF systems.

Parameter	System	
	NSF	KSF
Pugh's Ratio (B/G)	3.01	2.33
Cauchy Pressure *Pc* (GPa)	23.30	3.40
Kleinman's parameter	0.82	1.20
Universal Elastic Anisotropy	0.52	3.03
Chung-Buessem Anisotropy	0.00	0.20
Isotropic Poisson's Ratio	0.35	0.31
Longitudinal wave velocity v_l (m/s)	4800.685	4206.05
Transverse wave velocity v_t (m/s)	2303.318	2197.77
Average wave velocity v_m (m/s)	2589.820	2458.70
Debye temperature Θ_D (K)	325.10	292.50

Note S3.

We have already emphasized the importance of using a fractional particle occupancy scheme for the calculation convergence issues mentioned in Note S1. Three sets of fractional particle occupancy schemes are used to determine more suitable fractional particle occupancy schemes to reproduce the equilibrium geometric structure of the $^4T_2$ state and consequently the optical transition energies. The results of the calculated Mn^{4+}-F^- bond lengths and the volume of the $[MnF_6]^{2-}$ octahedron, as an index to characterize the distortion level of the excited states with respect to the ground state, at three sets of fractional particle occupancy schemes are given for KSF: Mn^{4+} in Table S9. Based on the obtained optimized geometric structures of the $^4A_2$ and $^4T_2$ states, we performed the HSE06 calculations to determine the excitation, emission and ZPL energies of $^4T_2$. The resulting values, together with the available experimental data, are summarized in Table S10. The calculated optical transition energies, predicated based on the geometric structure of the $^4T_2$ optimized by the first fractional particle occupancy scheme, show an overestimation of the Stokes shift energy, while an underestimation of the emission energy for the $^4A_2$-$^4T_2$ optical transition when compared to the experimental value of 2.676 eV [S11]. The reason for this discrepancy in the estimation of the $^4T_2 \rightarrow ^4A_2$ emission energy is due to an over-relaxation of the equilibrium geometric structure of the $^4T_2$ state by Scheme 1. The comparison between the

calculated and experimental value of $^4A_2$-$^4T_2$ optical transition energies shows better agreement with the experimental values when the geometric structure of the $^4T_2$ state is optimized with both Schemes 2 and 3. For the next calculation of geometry optimization of the $^4T_2$ state in Mn^{4+} doped NSF, NKSF and KSF, we have chosen Scheme 3. The reason for choosing Scheme 3 for predicting the energies of optical transitions becomes obvious, since the chosen particle occupations on the three t_{2g} KS orbitals [$\xi^{0.8}$ $\eta^{0.8}$ $\zeta^{0.4}$ θ^{1}] occupancy scheme corresponds to their energy distribution, since the t_{2g} KS orbitals of the Mn^{4+} ion in KSF splits into a doubly-degenerate E orbital and a singly-fold B_2 orbital.

Table S9. Calculated Mn^{4+}-F^- bond lengths (in Å) and volume V (in Å^3) of the $[MnF_6]^{2-}$ octahedron in KSF: Mn^{4+} in the $^4A_2$ ground and the $^4T_2$ excited states. Three sets of fractional particle occupancy schemes are used to calculate the equilibrium geometric structure of the $^4T_2$ excited state.

Parameter	State			
	$^4A_2$	$^4T_2$		
		Scheme 1	Scheme 2	Scheme 3
Mn-F^- 1×2	1.80833	1.78781	1.81130	1.79354
Mn-F^- 2×2	1.80931	1.87756	1.81436	1.87446
Mn-F^- 3×2	1.80932	1.90489	1.92615	1.88943
$V([MnF_6]^{2-})$	7.8930	8.5256	8.4400	8.4694

Table S10. The calculated excitation, emission, ZPL and Stokes shift energies(in eV) of the $^4A_2$-$^4T_2$ optical transition in KSF: Mn^{4+} based on the optimized geometric structures obtained at the SCAN level with three sets of fractional particle occupancy schemes.

Transition type	Calc.			Exp. [a]
	Scheme 1	Scheme 2	Scheme 3	
Excitation	2.7990	2.7990	2.7990	~2.7
Emission	2.4183	2.4759	2.4579	-

ZPL	2.7107	2.7297	2.6893	2.676
Stokes shift	0.3837	0.3230	0.3410	

[a] Ref. S11

Table S11. Calculated relative total electronic energies, (eV/cell) of Mn^{4+}-doped NSF.

Substituted site	Site symmetry	Total electronic energy	Relative total electronic energy
$Mn^{4+}\rightarrow Si$ (1)	D_3	-349.122800	0.124140
$Mn^{4+}\rightarrow Si$ (2)	C_3	-349.246940	0

Table S12. Calculated Mn^{4+}-F^- bond length (in Å), the volume (V) (in $Å^3$), the distortion index (D) (non-dimensional, × 10^{-3}) and the bond angle variation (σ^2) (in deg.^2) of $[MnF_6]^{2-}$ octahedron in the $^4A_2$ ground state and the ^2E and $^4T_2$ excited states of Mn^{4+} doped NSF, NKSF and KSF.

Systems	Parameter	State		
		$^4A_2$	^2E	$^4T_2$
NSF: Mn^{4+}	Mn-F¯1×1	1.80903	1.80656	1.79091
	Mn-F¯2×1	1.80913	1.80933	1.79235
	Mn-F¯3×1	1.80917	1.80985	1.88255
	Mn-F¯4×1	1.81201	1.81036	1.88433
	Mn-F¯5×1	1.81210	1.81315	1.89118
	Mn-F¯6×1	1.81242	1.81374	1.89346
	V	7.9116	7.9098	8.5055
	D	0.85	1.09	23.05
	σ^2	0.9674	0.9283	2.4126
NKSF: Mn^{4+}	Mn-F¯1×1	1.79569	1.79339	1.78823
	Mn-F¯2×1	1.79580	1.79771	1.80576
	Mn-F¯3×1	1.80653	1.80840	1.86361

	Mn-F¯4×1	1.80978	1.80890	1.86465
	Mn-F¯5×1	1.81191	1.81086	1.88977
	Mn-F¯6×1	1.82193	1.82238	1.89028
	V	7.8546	7.8549	8.4221
	D	4.21	4.20	19.24
	σ^2	3.5214	3.4020	5.6386
KSF: Mn^{4+}	Mn-F¯1×2	1.80833	1.80732	1.79354
	Mn-F¯2×2	1.80931	1.80974	1.87446
	Mn-F¯3×2	1.80932	1.81011	1.88943
	V	7.8930	7.8940	8.4694
	D	0.24	0.34	21.21
	σ^2	0.00000	0.00000	0.00000

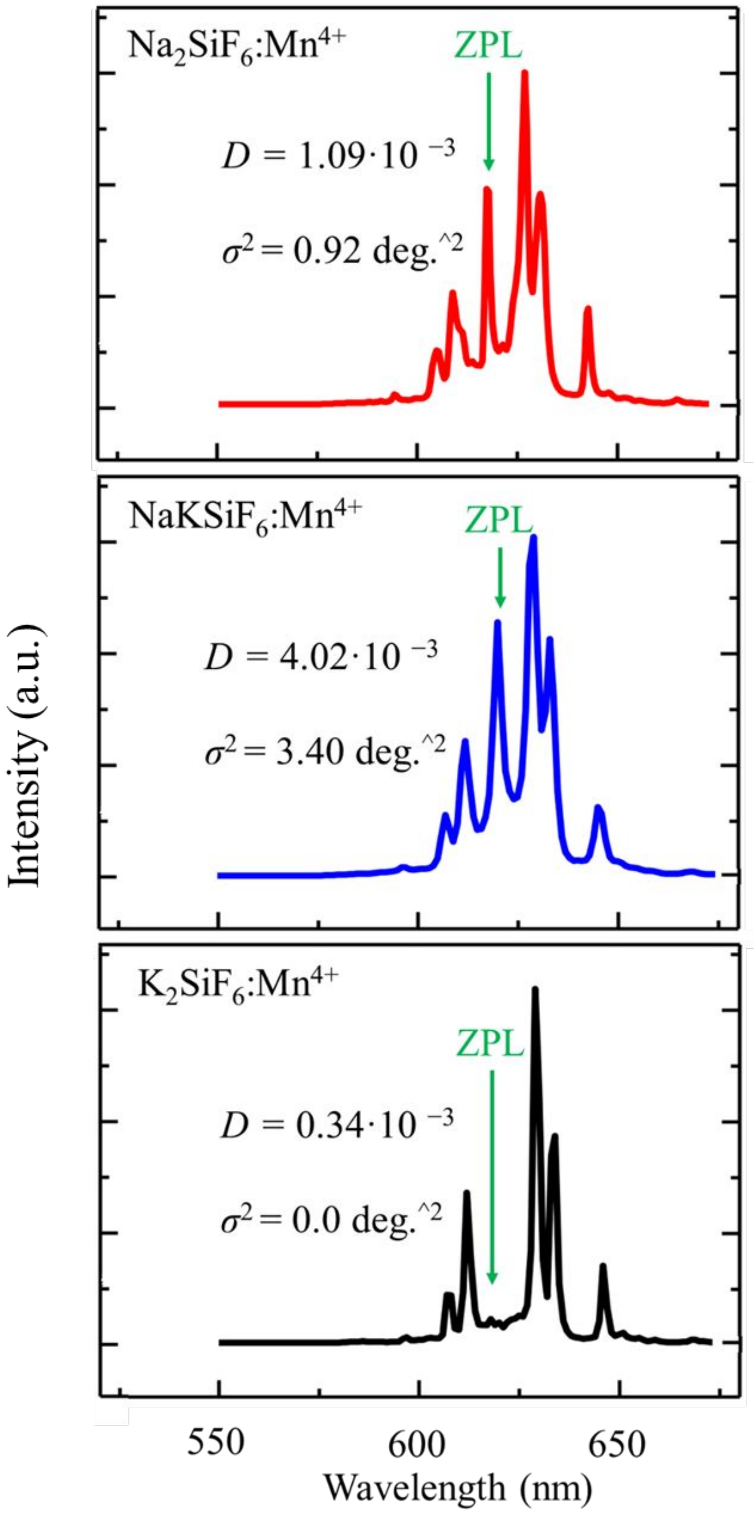

Fig. S1. The experimental photoluminescence spectra together with the calculated distortion index (D) and the bond angle variation (σ^2) of $[MnF_6]^{2-}$ octahedron in the ^2E state of Mn^{4+} doped NSF, NKSF and KSF phosphors. The experimental results are taken from Ref. S12.

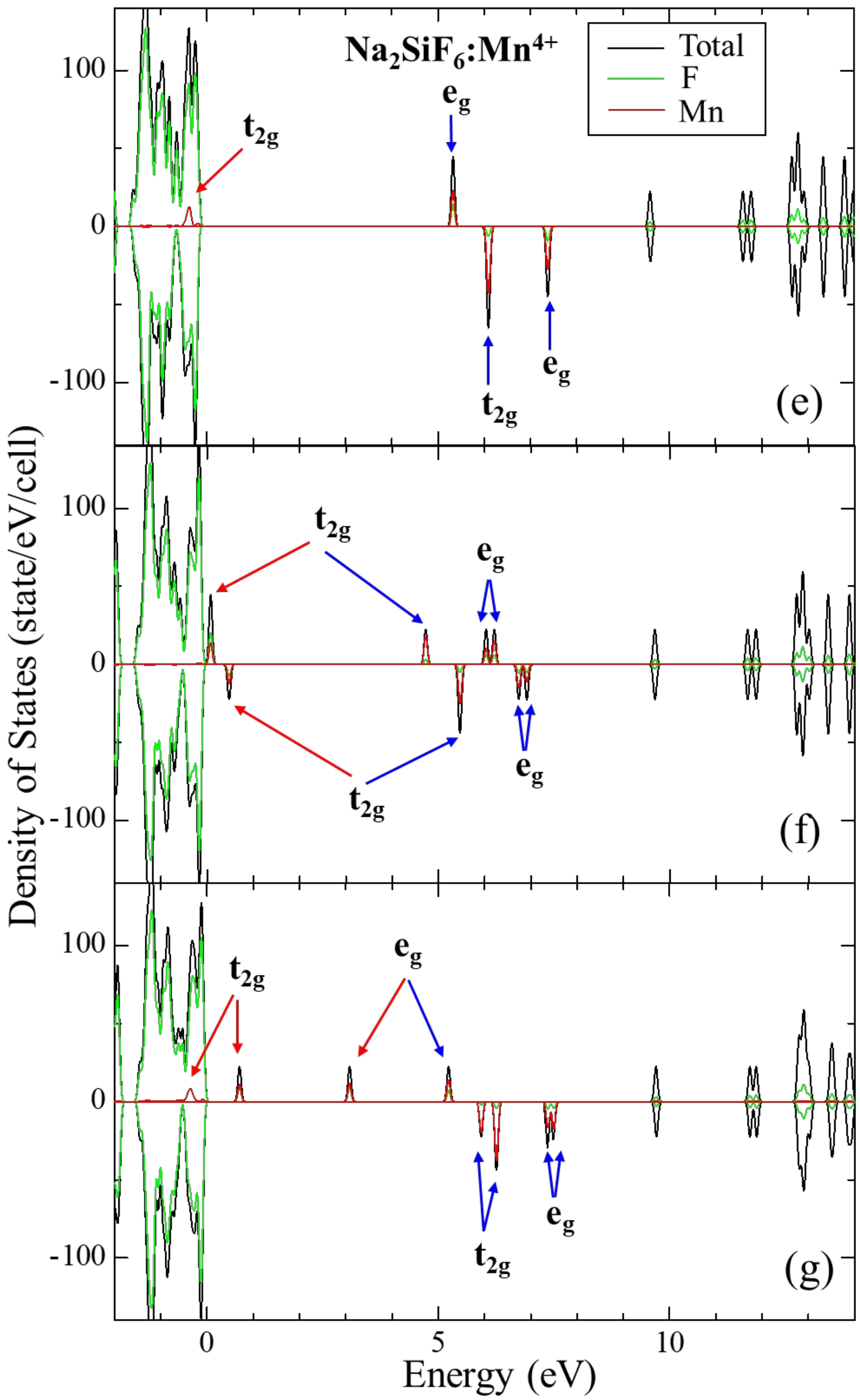
$Na_2SiF_6:Mn^{4+}$
Total
F
Mn
t_{2g}
e_g
(e)
(f)
(g)
Density of States (state/eV/cell)
Energy (eV)
100
0
-100
5
10

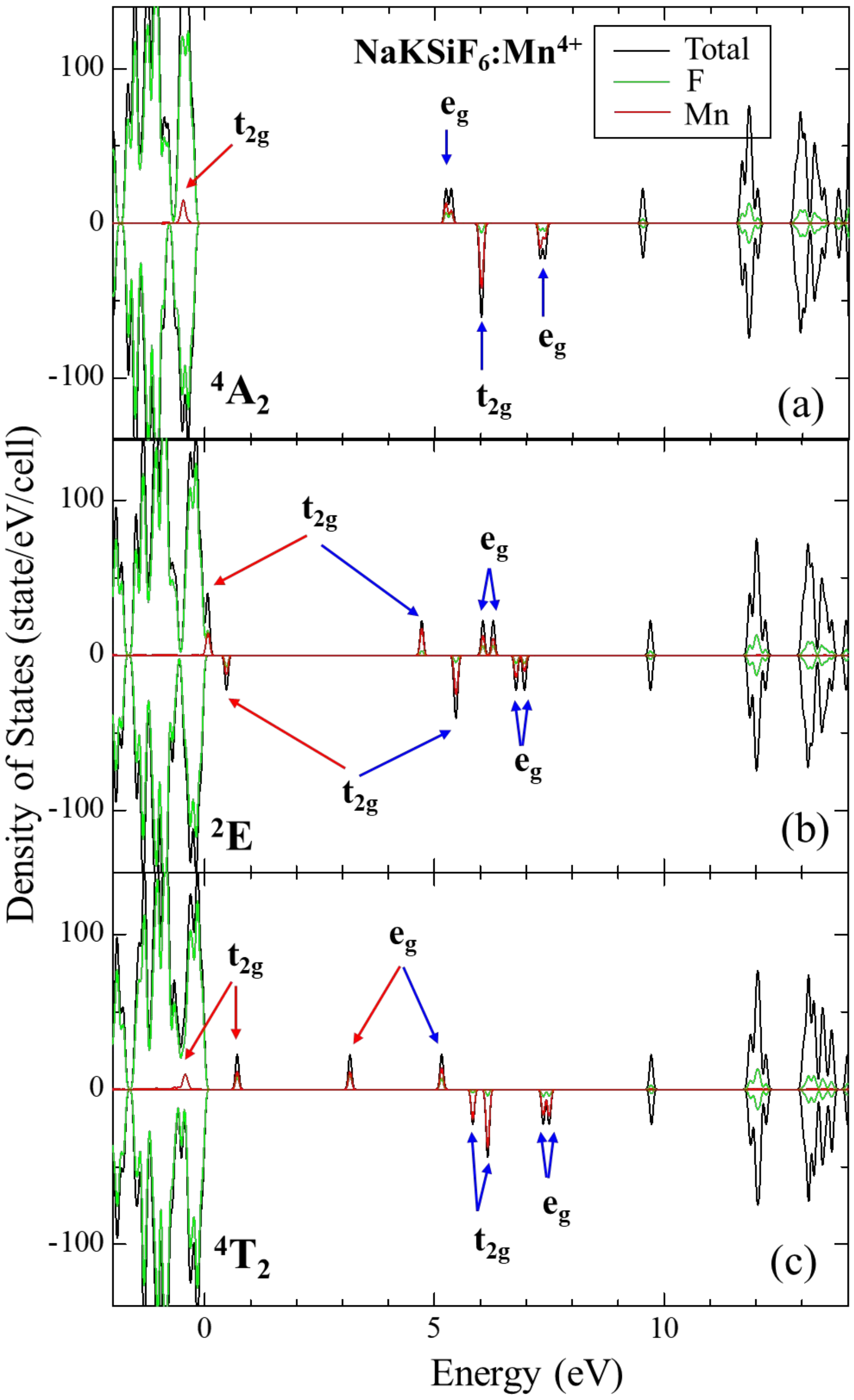

Fig. S2. Calculated electronic density of states of the ground state $^4A_2$ (a, d) and exited states ^2E (b, e) and $^4T_2$ (c, f) for NSF: Mn^{4+} and NKSF: Mn^{4+}, respectively. The occupied and unoccupied e_g and t_{2g} KS orbitals are represented by red and blue arrows, respectively.

References

S1. M.S. Kurboniyon, B. Lou, U. Zafari, F. Rahimi, A.M. Srivastava, T. Yamamoto, M.G. Brik, C.G. Ma. First-principles study of geometric and electronic structures, and optical transition energies of Mn^{4+} impurity ions: K_2SiF_6 as a prototype. J. Lumin. 263, 120103 (2023).

S2. W. Zou, B. Lou, M.S. Kurboniyon, M. Buryi, F. Rahimi, A.M. Srivastava, M.G. Brik, J. Wang, Ch.-G. Ma. Unraveling broadband near-infrared luminescence in Cr^{3+}-doped $Ca_3Y_2Ge_3O_{12}$ garnets: Insights from first-principles analysis. Materials 17, 1709 (2024).

S3. J.A.A. Ketelaar, Die Kristallstruktur von K-, Rb-, Cs- und Tl-Silicofluorid und von $LiMnO_4$-$3H_2O$, Z. für Kristallogr. Cryst. Mater. 92, 155-156 (1935).

S4. A.V. Gerasimenko, V.I. Sergienko, S.B. Ivanov, T.F. Antokhina. Crystal structure of $NaKSnF_6$. Koordinatsionnaya Khimiya (= Coordination Chemistry (USSR)) 18, 129-132 (1992).

S5. D. Babel. Structural chemistry of octahedral fluorocomplexes of the transition elements. In: Jørgensen, C.K., Neilands, J.B., Nyholm, R.S., Reinen, D., Williams, R.J.P. (eds) Structure and Bonding. Structure and Bonding, Springer, Berlin, Heidelberg, 1-87 (1967).

S6. J. Saaring, E. Feldbach, V. Nagirnyi, S. Omelkov, A. Vanetsev, and M. Kirm. Ultrafast Radiative Relaxation Processes in Multication Cross-Luminescence Materials. IEEE Trans. Nucl. Sci., 67, 6, 1009-1013 (2020).

S7. F. Mouhat, F.-X. Coudert. Necessary and sufficient elastic stability conditions in various crystal systems. Phys. Rev. B 90, 224104 (2014).

S8. W. Voigt. Wechselbeziehungen zwischen zwei Tensortripeln. (Elastizität und innere Reibung.). In: Lehrbuch der Kristallphysik. Vieweg+Teubner Verlag, Wiesbaden. 560-800 (1966).

S9. A. Reuss, Z. Angew. Calculation of the flow limits of mixed crystals on the basis of the plasticity of monocrystals. Math. Mech. 9, 49-58 (1929).

S10. R. Hill. The Elastic Behaviour of a Crystalline Aggregate. Proc. Phys. Soc. A 65, 349-354 (1952).

S11. T. Arai, S. Adachi. Excited states of $3d^3$ electrons in K_2SiF_6: Mn^{4+} red phosphor studied by photoluminescence excitation spectroscopy. Jpn. J. Appl. Phys. 50, 092401 (2011).

S12. Y. Jin, M.-H. Fang, M. Grinberg, S. Mahlik, T. Lesniewski, M.G. Brik, G.-Y. Luo, J. G. Lin, and R.-S. Liu. Narrow Red Emission Band Fluoride Phosphor $KNaSiF_6$: Mn^{4+} for Warm White Light-Emitting Diodes. ACS Appl. Mater. Interfaces 8, 11194-11203 (2016).
